# Plasma phosphorylated tau and neuropsychiatric symptoms in dementia with Lewy bodies

**DOI:** 10.1002/alz.14434

**Published:** 2024-12-28

**Authors:** Lucy L. Gibson, Maria C. Gonzalez, Nicholas J. Ashton, Diego Tovar‐Rios, Frédéric Blanc, Andrea Pilotto, Afina Lemstra, Claire Paquet, Clive Ballard, Henrik Zetterberg, Dag Aarsland, the European–Dementia With Lewy Bodies (E‐DLB) Consortium

**Affiliations:** ^1^ Centre for Healthy Brain Ageing Department of Psychological Medicine Institute of Psychiatry Psychology, and Neuroscience King's College London London UK; ^2^ Department of Quality and Health Technology Faculty of Health Sciences University of Stavanger Stavanger Norway; ^3^ The Norwegian Centre for Movement Disorders Stavanger University Hospital Stavanger Norway; ^4^ Centre for Age‐Related Medicine Stavanger University Hospital Stavanger Norway; ^5^ Department of Psychiatry and Neurochemistry Institute of Neuroscience and Physiology the Sahlgrenska Academy at the University of Gothenburg Göteborg Sweden; ^6^ Banner Alzheimer's Institute and University of Arizona St Phoenix Arizona USA; ^7^ Banner Sun Health Research Institute Sun City Arizona USA; ^8^ NIHR Biomedical Research Centre for Mental Health and Biomedical Research Unit for Dementia at South London and Maudsley NHS London UK; ^9^ Memory Resource and Research Centre Geriatrics Day Hospital Geriatrics Department University Hospital of Strasbourg Strasbourg France; ^10^ Neurology Unit Laboratory of Digital Neurology and Biosensors Neurobiorepository and Laboratory of advanced biological markers Department of Clinical and Experimental Sciences Italy and Department of continuity of care and frailty, Neurology Unit, ASST Spedali Civili of Brescia University of Brescia Brescia Italy; ^11^ Amsterdam Alzheimer Center Amsterdam University Medical Centers Amsterdam the Netherlands; ^12^ Université de Paris Neurology Center Assistance Publique Hôpitaux de Paris Lariboisière Fernand‐Widal Hospital Paris France; ^13^ Department of Clinical and Biomedical Sciences, Medical School University of Exeter Exeter UK; ^14^ Clinical Neurochemistry Laboratory Sahlgrenska University Hospital Göteborg Sweden; ^15^ Department of Neurodegenerative Disease Univeristy College London Institute of Neurology Queen Square London UK; ^16^ UK Dementia Research Institute at University College London London UK; ^17^ Hong Kong Center for Neurodegenerative Diseases Science Park Hong Kong China; ^18^ Wisconsin Alzheimer's Disease Research Center University of Wisconsin School of Medicine and Public Health University of Wisconsin‐Madison Madison Wisconsin USA

**Keywords:** dementia with Lewy bodies, hallucinations, neuropsychiatric symptoms, plasma biomarkers

## Abstract

**INTRODUCTION:**

Neuropsychiatric symptoms (NPSs) are common in dementia with Lewy bodies (DLB) but their neurobiological mechanisms are poorly understood.

**METHODS:**

NPSs and cognition were assessed annually in participants (DLB *n* = 222; Alzheimer's disease [AD] *n* = 125) from the European DLB (E‐DLB) Consortium, and plasma phosphorylated tau‐181 (p‐tau181) and p‐tau231 concentrations were measured at baseline.

**RESULTS:**

Hallucinations, delusions, and depression were more common in DLB than in AD and, in a subgroup with longitudinal follow‐up, persistent hallucinations and NPSs were associated with lower p‐tau181 and p‐tau231 in DLB. In adjusted linear mixed‐effects models, hallucinations at baseline were associated with greater longitudinal cognitive impairment in DLB, with a significant interaction with p‐tau231.

**DISCUSSION:**

Higher p‐tau181 and p‐tau231 levels were associated with a lower longitudinal risk of NPSs and hallucinations in early‐stage DLB. However, the interaction between hallucinations and p‐tau231 suggests that when AD co‐pathology and hallucinations do co‐exist in DLB that they may synergistically exacerbate cognitive decline.

**Highlights:**

- Neuropsychiatric symptoms (NPSs) were more common in dementia with Lewy bodies (DLB) than in Alzheimer's disease (AD).
- Lower plasma phosphorylated tau‐231 (p‐tau231) and p‐tau181 levels were associated with persistent hallucinations in DLB.
- Lower plasma p‐tau231 and p‐tau181 levels were associated with an increased risk of persistent NPSs in early DLB.
- Hallucinations at baseline were associated with greater cognitive dysfunction in DLB, and there was an interaction with p‐tau231.

## 1 BACKGROUND

Neuropsychiatric symptoms (NPSs) such as hallucinations, delusions, depression, agitation, and apathy are highly prevalent in neurodegenerative dementias and are particularly common in dementia with Lewy bodies (DLB).^1^, ^2^, ^3^, ^4^ The clinical implications of NPSs are manifold, including increased caregiver burden and distress, higher mortality, accelerated disease progression, and worsened quality of life.^3^, ^4^, ^5^, ^6^, ^7^, ^8^, ^9^, ^10^, ^11^ However, despite the importance of NPSs for patients and their caregivers, few effective treatment options currently exist, and a better understanding of the neurobiological correlates and the mechanisms driving NPSs is needed to facilitate drug development.^12^

Increasingly, evidence suggests that NPSs are intrinsic to the neurodegenerative process and can occur in the earliest stages of disease.^13^, ^14^, ^15^, ^16^ Although DLB is defined neuropathologically by the presence of aggregated phosphorylated α‐synuclein‐forming Lewy bodies (LBs), the inherent neuropathological heterogeneity is now well established and co‐morbid Alzheimer's disease neurodegenerative change (ADNC) is found in more than 50% of postmortem cases.^17^, ^18^ The presence of AD co‐pathology in DLB has clinical implications including accelerated cognitive decline and reduced life expectancy.^19^, ^20^, ^21^, ^22^, ^23^, ^24^, ^25^, ^26^, ^27^, ^28^ There may also be implications for NPSs; postmortem studies in both AD and DLB suggest that agitation, depression, and psychosis increase proportionately to the spread of tau and neurofibrillary tangles and additional co‐pathologies may cumulatively increase the burden of NPSs.^29^, ^30^, ^31^, ^32^

However, postmortem studies mostly capture end‐stage disease, and in vivo studies are needed to explore the influence of neuropathological changes earlier in the disease. In both AD and cognitively unimpaired individuals, positron emission tomography (PET) and cerebrospinal fluid (CSF) studies find NPSs to be associated with amyloid beta (Aβ) and tau pathologies, supporting an etiological role for ADNC in the development of these symptoms, although results are variable.^33^, ^34^, ^35^, ^36^, ^37^, ^38^, ^39^, ^40^, ^41^ Newly established blood‐based biomarkers offer cost‐effective and noninvasive alternatives to measure changes in AD co‐pathology in vivo.^42^, ^43^ Plasma tau phosphorylated at threonine 181 and/or 231 (p‐tau181 and p‐tau231, respectively) are specific for AD pathology and lie on a continuum, with p‐tau231 more responsive at lower thresholds of Aβ pathology before there is global spread, whereas p‐tau181 shows greater sensitivity to a higher burden of Aβ.^44^, ^45^, ^46^, ^47^, ^48^, ^49^ These plasma biomarkers also have clinical utility in DLB, showing good correlation with both AD CSF biomarkers and PET imaging in DLB, and are most elevated in DLB cases with AD co‐pathology post‐mortem.^50^, ^51^, ^52^

In the prodromal stages of AD, increased plasma p‐tau181 level is associated with a greater burden of NPSs, but few studies have explored in vivo associations in DLB.^15^ Several cross‐sectional CSF studies suggest that the presence of ADNC in DLB may be associated with a lower prevalence of core features, including hallucinations, but the results are inconsistent and this has not been explored for other NPSs.^25^, ^28^, ^53^, ^54^ One study found no difference in the frequency of visual hallucinations at baseline in patients with DLB stratified by plasma p‐tau181 concentration^54^ and recent studies in AD have highlighted the importance of longitudinal studies to differentiate persistent NPS from transient, reactive symptoms, which are less likely to be related directly to neuropathological changes.^15^, ^37^ From the preclinical stages of AD and Parkinson's disease (PD), the presence of NPSs is associated with increased cognitive impairment and accelerated decline,^55^, ^56^, ^57^, ^58^ but the mechanisms driving this are unknown and it is not clear to what extent co‐morbid neuropathology modulates these changes. Studies in DLB are lacking; however, one recent study found no association between NPSs and prospective cognitive decline in the prodromal stages.^59^

We aimed to investigate the cross‐sectional and longitudinal associations between NPSs and plasma markers of p‐tau in DLB and AD, and to explore how AD co‐pathology may contribute to the inherent clinical heterogeneity associated with NPSs. The presence of NPSs and AD co‐pathology has been independently associated with worse cognitive trajectories in DLB^60^ and here we aimed to investigate whether they have separate or synergistic effects on cognition in DLB. We also aimed to investigate the potential of p‐tau181 and p‐tau231 as prognostic biomarkers associated with NPSs in DLB and AD.

## 2 METHODS

### 2.1 Participants

Participants were included from the European DLB (E‐DLB) Consortium, a multicenter international initiative recruiting patients, which has been described previously.^61^ Five centers (Amsterdam, Brescia, Paris, Strasbourg, and Stavanger) contributed to this study, with participants enrolled between February 2006 and September 2020. Participants included patients with clinically diagnosed AD (*n* = 125) and probable DLB (*n* = 222).

In the Consortium, probable DLB is diagnosed using the International Consensus Criteria, and core diagnostic features are evaluated (fluctuating cognition, rapid eye movement [REM] sleep abnormalities, parkinsonism, and recurrent visual hallucinations).^62^ In 77 patients (34.7%), diagnosis of probable DLB was supported by an abnormal dopamine transporter single‐photon emission computed tomography (DaT‐SPECT) scan. AD cases were defined according to the International Classification of Diseases, Tenth Revision (ICD‐10) criteria.^63^

Participants are referred from outpatient clinics including neurology, psychiatry, geriatric medicine, movement disorders, and memory clinics. Exclusion criteria included acute delirium, severe physical conditions that would impact study participation, and previous major psychiatric or neurological disorders.

The use of these data and the international analysis of samples were approved by the local ethics committee in each participating center, and the steering committee of the E‐DLB. Participants gave written consent for use of their de‐identified clinical and biomarker data.

### 2.2 Clinical assessment

Demographic data were collected including age, sex, years of education, and duration of symptoms from the first motor or cognitive symptom (see Table 1). Global cognitive function was assessed using the Mini‐Mental State Examination (MMSE). A binary assessment (present or absent) of each NPS (hallucination, delusion, depressive symptom, apathy, agitation, or any NPS) was included as the outcome measure.

**TABLE 1 alz14434-tbl-0001:** Baseline characteristics of the cohort.

	DLB (*n* = 222)	AD (*n* = 125)	*p*
Demographic characteristics			
Age, years	70.9 (8.4)	71.4 (8.0)	0.806
Female, no. (%)	103 (46.4)	67 (53.6)	0.219
Education, years^a^	10.0 (4.0)	11.4 (3.4)	**0.034**
Duration of disease, years^b^	2.1 (2.7)	1.7 (2.0)	**0.036**
MMSE score	23.2 (5.3)	22.6 (4.9)	0.142
Neuropsychiatric symptoms (NPSs)			
Hallucinations, no. (%)	112 (53.3)	14 (12.0)	**< 0.001**
Visual hallucinations or illusions, (%)^c^	53 (51.5)	9 (13.2)	**< 0.001**
Auditory hallucinations, no (%)^c^	16 (15.5)	0	**< 0.001**
Olfactory hallucinations, no. (%)^c^	16 (15.7)	2 (2.9)	**0.010**
Delusions, no. (%)	43 (22.4)	12 (10.6)	**0.013**
Depression, no. (%)	79 (43.2)	31 (28.2)	**0.013**
Agitation, no. (%)^d^	22 (21.2)	18 (36.7)	0.050
Apathy, no. (%)^d^	52 (50.5)	27 (55.1)	0.607
Any NPS, no. (%)	171 (81.4)	55 (46.6)	**< 0.001**
Plasma biomarker			
Plasma p‐tau181 level, pg/mL	18.0 (10.1)	21.4 (8.6)	**< 0.001**
Plasma p‐tau231 level, pg/mL	12.5 (6.6)	14.9 (6.3)	**< 0.001**

*Note*: The data reported are mean (SD) unless frequency (%) is reported as indicated in the table.

Bold indicates significance *p*<0.05.

Abbreviations: AD, Alzheimer's disease; DLB, dementia with Lewy bodies; HC, healthy control; MMSE, Mini‐Mental State Exam; NPS, neuropsychiatric symptom.

^a^ Assessed in three centers: *n* = 108 DLB, *n* = 48 AD.

^b^ Assessed in four centers: *n* = 193 DLB, *n* = 101 AD.

^c^ Assessed in one center: *n* = 103 DLB, *n* = 68 AD.

^d^ Assessed in four centers *n* = 103 DLB, *n* = 49 AD.

In four centers, NPSs were assessed in the Neuropsychiatric Inventory (NPI) and the presence of each NPS was defined by a score ≥1 in the relevant item.^64^ One center did not use the NPI in the assessment of NPSs and evaluated psychotic symptoms with the Fénelon scale, providing a fine‐grained assessment of hallucinations and delusions. The presence of any hallucination or delusion subtype in the scale was required for a binary assessment of hallucinations or delusions, respectively.^65^ In this center, affective symptoms were assessed in the Mini‐International Neuropsychiatric Interview 5.0.0 (MINI). Depression was classified by score ≥2, where one point was derived from the low mood or anhedonia item.^66^ For the purposes of this study, the presence of “any NPS” was defined by positive assessment of hallucinations, delusions, depression, apathy, or agitation.

**RESEARCH IN CONTEXT**

1. **Systematic review**: The presence of Alzheimer's disease (AD) co‐pathology in dementia with Lewy bodies (DLB) is common and has implications for the clinical phenotype. In postmortem studies, AD co‐pathology is associated with an increased burden of neuropsychiatric symptoms (NPSs), but this has not been replicated in vivo.
2. **Interpretation**: In DLB, higher levels of p‐tau181 and p‐tau231 were associated with a lower risk of NPSs and hallucinations, indicating that AD co‐pathology is not a primary driver of NPSs in the early stages of DLB. Hallucinations and increased plasma p‐tau231 level interact in association with cognitive decline in DLB, suggesting that when they co‐exist they are particularly detrimental to cognition.
3. **Future directions**: Identifying the neural correlates of NPSs in neurodegenerative disease is critical to drive the development of future treatment options. Studies in AD with novel seed amplification assays of α‐synuclein can further explore associations with Lewy body pathology with NPSs, whereas additional positron emission tomography (PET) studies are needed to localize interactions.

### 2.3 Longitudinal assessment

A subgroup of included participants (DLB *n* = 172; AD *n* = 83) had up to two neuropsychiatric assessments after baseline (median 3 assessments, range 2–3). NPSs were considered to be persistent when they were present across at least two assessments. Global cognition was assessed annually for up to 5 years with a follow‐up duration mean (SD) of 2.59 ± 1.46 years.

### 2.4 Phosphorylated tau blood measurements

Plasma samples were collected at baseline in each participating centre. Plasma p‐tau181 and p‐tau231 concentrations were measured centrally using in‐house single molecule array (Simoa) methods on the HD‐X (Quanterix)[Table alz14434-tbl-0001] at the Clinical Neurochemistry Laboratory, Sahlgrenska University Hospital (Mölndal, Sweden) as reported previously.^49^, ^67^ Analysts were blinded to clinical data. The analytical variation of the internal quality control (iQC) samples for p‐tau181 was <10% across the whole cohort. Plasma values under the lower limit of quantification (LLOQ) were excluded. All measurements were performed using one batch of reagents and samples were randomized.

### 2.5 Statistical analysis

The cohort's baseline characteristics were grouped according to AD and probable DLB. Descriptive statistics are presented as means (SDs) for continuous variables and counts (percentages) for categorical variables. Baseline comparisons were made using the Kruskal–Wallis test for continuous variables and the Fisher exact test for categorical variables. Hallucinations, delusions, depression, agitation, and apathy were selected as primary outcomes due to their clinical relevance in DLB.

For modeling purposes, the measurements of p‐tau181 and p‐tau231 were centered and scaled to be interpreted as SDs. To account for differences in the assessment of NPSs in one center, the center variable was dichotomized. The association between p‐tau181 and p‐tau231 and the presence of each NPS at baseline was assessed using independent logistic regression models, adjusted for age, sex, duration of symptoms, baseline MMSE score, and the dichotomized center. Robust estimation of standard errors was used to account for data clustering and results were presented as odds ratios (ORs). Analyses were conducted across clinical groups of AD and probable DLB.

We also evaluated the association between plasma biomarker measurements and the longitudinal presence of symptoms using a multinomial Logistic Regression Model with persistent and single‐episode NPSs compared to a reference group with no NPSs. These models were adjusted for age, sex, duration of symptoms, baseline MMSE score, and center dichotomized. Robust estimation of standard errors was used to account for data clustering and results were presented as relative risk ratios (RRRs). Analyses were conducted across clinical groups of AD and probable DLB. Sensitivity analyses were conducted within clinical groups for early (< 2 years disease onset, *n* = 197; 128 DLB, 69 AD; mean 0.49 years ± 0.53) and late (>2 years disease onset, *n* = 97; 65 DLB, 32 AD; mean 4.76 years ± 2.54) phases of the disease.

In addition, logistic regression was performed to evaluate the odds of future or incident NPSs with p‐tau181 and p‐tau231 as the predictor variables adjusted for age, disease duration, sex, cognition, and dichotomized center. The models for future NPSs were performed for all patients with longitudinal follow‐up, whereas only those patients without the baseline NPSs were included in the model for incident NPSs.

Finally, in independent linear mixed models (LMMs) we explored the association between longitudinal decrease in MMSE score and the baseline presence of hallucinations with potential interactions with p‐tau181 or p‐tau231. Each model included random intercepts and slopes for each participant to account for individual variability and the correlation of repeated measures over time. Models were adjusted for potential confounders, namely age and sex, and included the interactions between time and hallucinations and the interaction between p‐tau and hallucinations. A sub‐analysis included years of education as a covariate for participants for whom these data were available. For modeling purposes, the MMSE score was transformed using the square root of (30 − MMSE score). This transformation inverts the interpretation of the coefficients so that positive estimations reflect worse cognitive performance. The estimated effect of each plasma biomarker on longitudinal cognitive function for those with and without NPSs at baseline was calculated as a linear combination of the coefficients estimated in the model. Results are reported as 95% confidence intervals (CIs), and significance was established at 5%. All analyses were performed using Stata version 17.0, and graphs were created with R version 4.2.3.

## 3 RESULTS

### 3.1 Demographic characteristics

The baseline characteristics of the 347 included participants are presented in Table 1. There were no differences between the DLB and AD group in terms of age, sex, or baseline cognition, but the DLB group had a longer duration of symptoms and fewer years of education. Patients with DLB had lower p‐tau181 and p‐tau231 concentrations relative to the AD group (see Table 1).

### 3.2 Difference in NPSs across clinical groups

Patients with DLB were more likely to have NPSs at baseline. These data are presented in detail in Table 1. Hallucinations were more common in patients with DLB, and in the center where hallucination subtypes were recorded (*n* = 103 DLB and *n* = 68 AD), visual, auditory, and olfactory hallucinations were significantly more common in DLB than in AD. In this center, 82% (*n* = 53) of hallucinations reported in DLB were visual, whereas all hallucinations reported by patients with AD were visual in nature, albeit for a significantly smaller overall number (*n* = 9; 13%).

The subgroup followed longitudinally (with median 3 assessments of NPSs, range 2–3) were demographically similar (see Table S1) and patients with DLB had a significantly higher frequency of persistent NPSs than the patients in the AD group. Persistent hallucinations, visual hallucinations illusions, depression, and delusions were more common in patients with DLB than with AD (see Table 2).

**TABLE 2:** Longitudinal neuropsychiatric symptom by clinical group.

	DLB (*n* = 172)				AD (*n* = 83)				
Symptom, no. (%)	*N*	None	Single episode	Persistent (2+)	*n*	None	Single episode	Persistent (2+)	*p*
Hallucinations	172	52 (30.2)	34 (19.8)	86 (50.0)	83	62 (74.7)	15 (18.1)	6 (7.2)	**< 0.001**
VH or illusions	103	32 (31.1)	25 (24.3)	44 (44.7)	67	49 (73.1)	13 (19.4)	5 (7.5)	**< 0.001**
Delusions	154	97 (63.0)	29 (18.8)	28 (18.2)	73	60 (82.2)	10 (13.7)	3 (4.1)	**0.003**
Depression	149	67 (45.0)	34 (22.8)	48 (32.2)	70	45 (64.3)	17 (24.3)	8 (11.4)	**0.002**
Apathy	65	28 (43.1)	11 (16.9)	26 (40.0)	12	4 (33.3)	3 (25)	5 (42.7)	0.716
Agitation	68	48 (70.6)	9 (13.2)	11 (16.2)	12	6 (50.0)	3 (25.0)	3 (25.0)	0.322
Any NPS	172	17 (9.9)	33 (19.2)	122 (70.9)	83	42 (50.6)	28 (33.7)	13 (15.7)	**< 0.001**

Abbreviations: AD, Alzheimer's disease; DLB, dementia with Lewy bodies; NPS: neuropsychiatric symptom; VH, visual hallucinations.

*Note*: Bold denotes significance at *p*<0.05.

### 3.3 Cross‐sectional association of NPSs and plasma biomarkers in AD and DLB

Increased concentration of plasma p‐tau231 and p‐tau181 was associated with lower odds of visual hallucinations or illusions at baseline in DLB (p‐tau231 OR 0.59, 95% CI 0.36–0.97, *p* = 0.038; p‐tau181 OR 0.38, 95% CI 0.21–0.70, *p* = 0.020). However, there was no association between hallucinations overall and p‐tau231 or p‐tau181 cross‐sectionally at baseline. In addition, there was no association between hallucinations and p‐tau231 or p‐tau181 in AD. (See Table S2.) The presence of NPSs overall was not associated with p‐tau181 or p‐tau231 at baseline in DLB or AD.

In sensitivity analysis restricted to patients with new‐onset DLB or duration of disease <2 years (*n* = 117), increased p‐tau181 and p‐tau231 were associated with lower odds of any NPS at baseline (p‐tau181 OR 0.54, 95% CI 0.35–0.85, *p* = 0.007; p‐tau231 OR 0.57, 95% CI 0.36–0.92, *p* = 0.020). In the early phase of the disease, increased p‐tau181 was also associated with lower odds of hallucinations at baseline (OR 0.54, 95% CI 0.32–0.92, *p* = 0.023), but p‐tau231 was not associated. In patients with DLB and disease duration ≥2 years (*n* = 58), there was no association between p‐tau181 and p‐tau231 and any NPSs or hallucinations at baseline.

In logistic regression adjusted for age, sex, cognition, center, and symptom duration, increased p‐tau181 in AD was associated with a higher risk of delusions (OR 2.05, 95% CI 1.11–3.78, *p* = 0.022) and symptoms of depression (OR 2.06, 95% CI 1.17–3.63, *p* = 0.012), but p‐tau231 was not associated (see Table S2).

### 3.4 Association between phosphorylated tau and longitudinal NPSs in DLB

**FIGURE 1:**
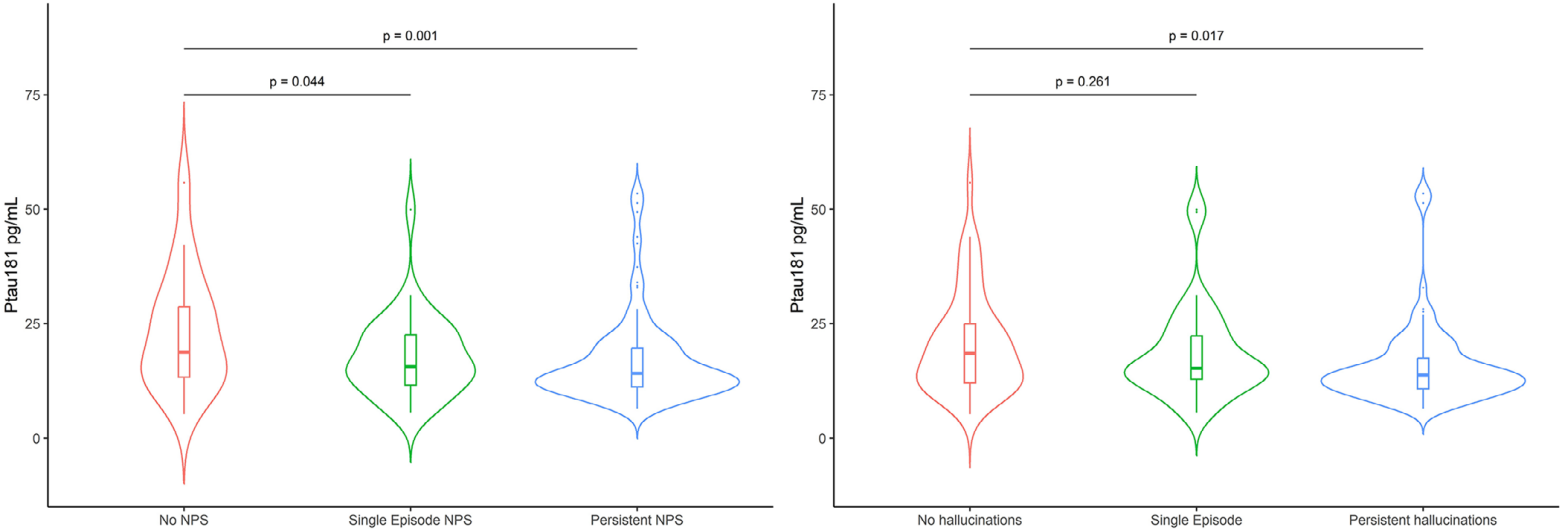
Violin plot of baseline plasma p‐tau181 longitudinal NPSs and hallucinations in DLB. Persistent NPSs and hallucinations were associated with reduced concentrations of p‐tau181 in adjusted multinomial logistic regression illustrated in these plots. DLB, dementia with Lewy bodies; NPSs, neuropsychiatric symptoms.

**FIGURE 2:**
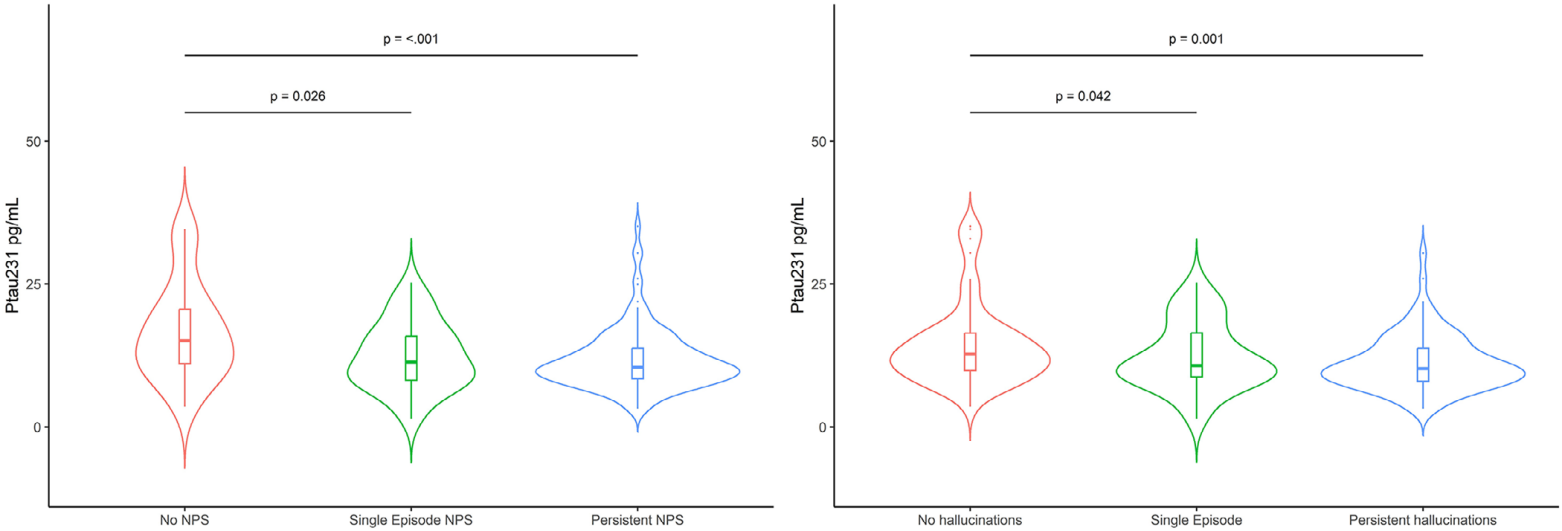
Violin plot of baseline plasma p‐tau231 longitudinal NPSs and hallucinations in DLB. Persistent NPSs and hallucinations were associated with reduced concentrations of p‐tau231 in adjusted multinomial logistic regression illustrated in these plots. DLB, dementia with Lewy bodies; NPSs, neuropsychiatric symptoms.

In patients with DLB, higher plasma p‐tau181 and p‐tau231 concentrations were associated with a decreased risk of both single‐episode and persistent NPSs (see Table 3 and Figure 1). Increased plasma p‐tau181 and p‐tau231 concentrations were also associated with a reduced risk of persistent hallucinations and visual hallucinations or illusions specifically (Table 3 and Figures 1, 2). The significance and direction of results were unchanged in sensitivity analysis restricted to patients with DLB and duration of disease <2 years (*n* = 102). However, there was no association between p‐tau181 and p‐tau231 and persistent hallucinations or NPSs in patients with DLB and duration of disease ≥2 years (*n* = 40).

**TABLE 3:** Association between longitudinal neuropsychiatric symptoms and plasma p‐tau181 and p‐tau231 respectively in DLB.

	*p‐tau181*			*p‐tau231*		
Symptom	RRR	95% CI	*p*‐value	RRR	95% CI	*p*‐value
**Hallucinations**						
No NPS	1.00	Reference level		1.00	Reference level	
Single episode	0.78	0.50–1.20	0.261	0.52	0.28–0.98	**0.042**
Persistent (2+)	0.55	0.34–0.90	**0.017**	0.45	0.29–0.72	**0.001**
**VH or illusions** ^a^						
No NPS	1.00	Reference level		1.00	Reference level	
Single episode	0.92	0.52–1.62	0.780	0.70	0.36–1.39	0.309
Persistent (2+)	0.30	0.14–0.63	**0.002**	0.48	0.28–0.80	**0.006**
**Delusions**						
No NPS	1.00	Reference level		1.00	Reference level	
Single episode	1.26	0.87–1.81	0.220	1.42	0.82–2.47	0.212
Persistent (2+)	1.12	0.68–1.84	0.656	0.97	0.52–1.81	0.919
**Depression**						
No NPS	1.00	Reference level		1.00	Reference level	
Single episode	1.40	0.89–2.21	0.144	1.26	0.70–2.26	0.449
Persistent (2+)	1.27	0.83–1.95	0.266	1.35	0.84–2.18	0.214
**Agitation** ^b^						
No NPS	1.00	Reference level		1.00	Reference level	
Single episode	0.48	0.19–1.23	0.127	0.68	0.24–1.89	0.455
Persistent (2+)	1.04	0.48–2.28	0.919	1.28	0.49–3.33	0.616
**Apathy** ^b^						
No NPS	1.00	Reference level		1.00	Reference level	
Single episode	1.51	0.74–3.06	0.256	1.88	0.67–5.25	0.227
Persistent (2+)	1.64	0.75–3.58	0.218	0.43	0.18–1.02	0.056
**Any NPS**						
No NPS	1.00	Reference level		1.00	Reference level	
Single episode	0.51	0.27–0.98	**0.044**	0.44	0.21–0.91	**0.026**
Persistent (2+)	0.38	0.21–0.67	**0.001**	0.31	0.17–0.59	**< 0.001**

*Note*: RRR in adjusted logistic regression.

Abbreviations: DLB, dementia with Lewy bodies; NPS: neuropsychiatric symptom; RRR, relative risk ratio; VH, visual hallucinations.

Bold denotes significance at *p*<0.05.

^a^ Only measured in one center missing *n* = 57.

^b^ Not assessed in one center missing *n* = 85.

### 3.5 Biomarker predictions of NPSs in DLB

In DLB, in patients with longitudinal follow‐up, hallucinations were present at baseline in 54.7% (*n* = 94) and in 60.5% (*n* = 104) in the 2 years after baseline. Lower concentrations of plasma p‐tau231 and p‐tau181 in DLB were associated with an increased odds of future hallucinations, irrespective of baseline symptomatic status (p‐tau231 OR 0.50, 95% CI 0.33–0.76, *p* = 0.001; p‐tau181 OR 0.68, 95% CI 0.47–0.98, *p* = 0.037). In addition, in patients without hallucinations at baseline, lower p‐tau231 concentrations were associated with an increased risk of incident new‐onset hallucinations in the following 2 years (OR 0.4, 95% CI 0.19–0.96, *p* = 0.039). There was no association with p‐tau181.

NPSs occurred in 77.9% (*n* = 134) of patients with DLB at follow‐up and increased baseline p‐tau231 or p‐tau181 was associated with reduced odds of future NPSs in DLB (ptau231 OR 0.46, 95% CI 0.29–0.75, *p* = 0.002; p‐tau181 OR 0.60, 95% CI 0.39–0.93, *p* = 0.023). In DLB patients without NPSs at baseline (*n* = 39, 18.6%), 55.3% (*n* = 21) developed NPSs in the following 2 years, and in this smaller subpopulation increased baseline p‐tau231 was associated with a reduced odds of incident NPSs (p‐tau231 OR 0.39, 95% CI 0.16–0.97, *p* = 0.042) but there was no association with p‐tau181. (See Table S2.)

### 3.6 Association between NPS and cognition in DLB

**FIGURE 3:**
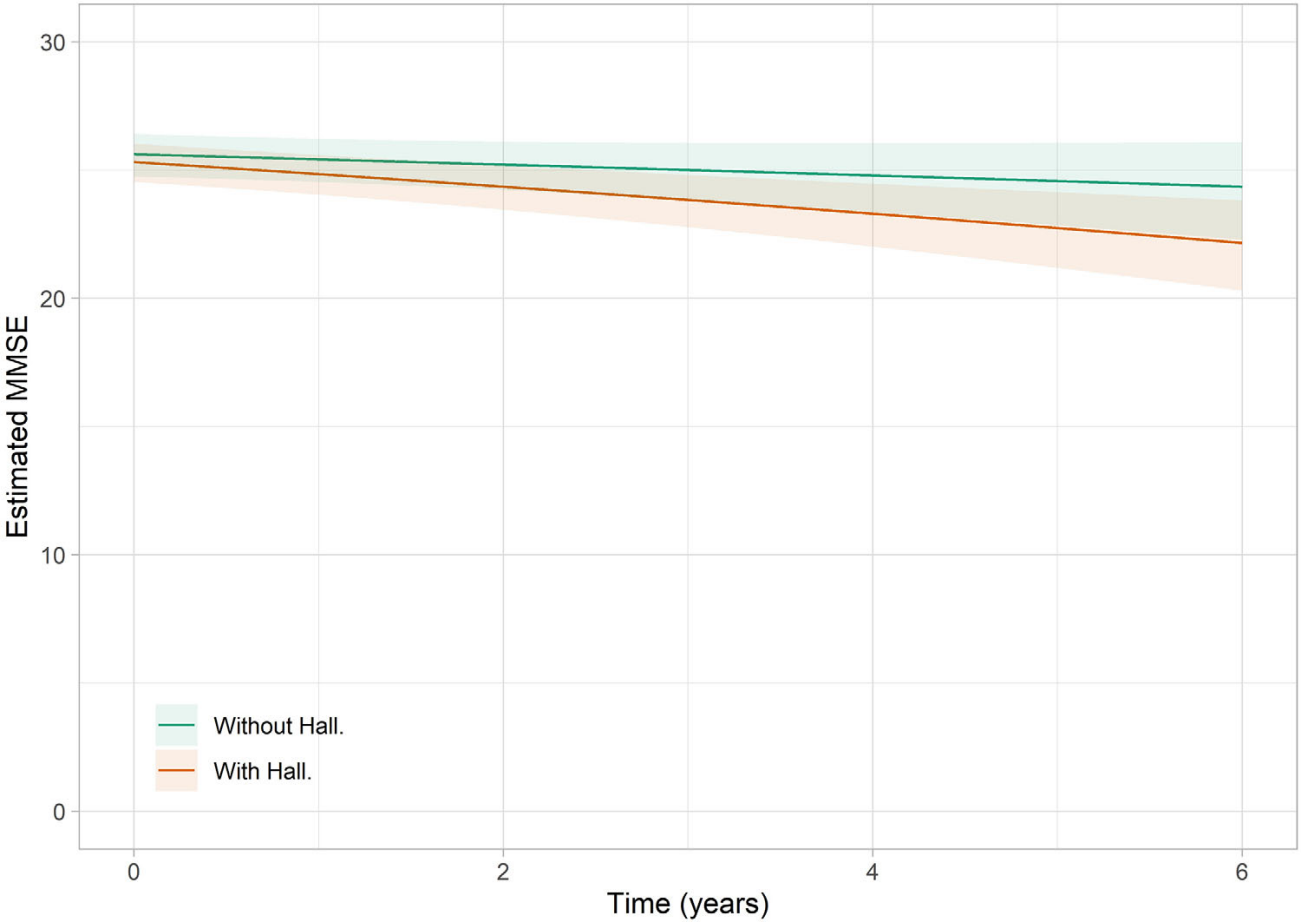
Decline in MMSE over time in patients with or without hallucinations at baseline in DLB. DLB, dementia with Lewy bodies; MMSE, Mini‐Mental State Examination.

**FIGURE 4:**
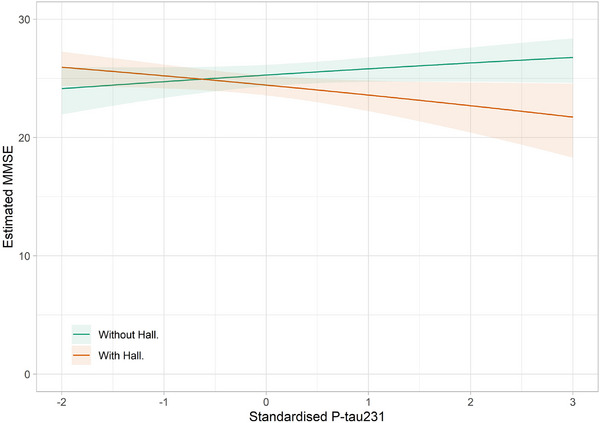
Interaction between p‐tau231 and the presence of hallucinations at baseline on cognitive function in patients with DLB. DLB, dementia with Lewy bodies.

In the longitudinal LMM for DLB, there was no statistically significant difference in the MMSE score at baseline between those with hallucinations and those without (*β* = 0.08, 95% CI −0.17 to –0.33, *p* = 0.519). However, in this model, the presence of hallucinations at baseline was associated with significantly lower cognitive scores longitudinally (EST  0.11, 95% CI 0.06–0.16, *p* < 0.001), but there was not statistically significant difference in the rate of cognitive decline between those with and without hallucinations at baseline (*β* = 0.06, 95% CI −0.02 to 0.14, *p* = 0.156) (see Figure 3). Higher levels of p‐tau231 were also associated with lower cognitive scores (EST 0.17, 95% CI 0.01–0.34, *p* = 0.041), and there was a significant interaction between p‐tau231 and hallucinations (*β* = 0.30, 95% CI 0.08–0.52, *p* = 0.013) (see Figure 4). This indicates that cognitive decline is more pronounced in individuals with higher p‐tau231 level and hallucinations and that the combined effect of increased p‐tau231 and hallucinations is particularly detrimental to cognitive function.

Furthermore, when the model was additionally adjusted for years of education (reported for a subgroup, *n* = 93), cognitive decline in individuals with hallucinations at baseline was significantly faster than in those without hallucinations (*β* = 0.30, 95% CI 0.08–0.52], *p* = 0.008), and the interaction with p‐tau231 remained significant (*β* = 0.32, 95% CI 0.01 to 0.64, *p* = 0.044).

However, in a similar model for p‐tau181, the MMSE scores were not significantly different between those with and without hallucinations at baseline (*β* = 0.09, 95% CI −0.17 to 0.35, *p* = 0.494). Furthermore, there was no significant interaction between p‐tau181 levels and the presence of hallucinations on cognitive decline (*β* = 0.09, 95% CI −0.15, 0.33, *p* = 0.440), even after adjusting for years of education.

## 4 DISCUSSION

In patients with recently diagnosed DLB and AD, we investigated cross‐sectional and longitudinal associations between NPSs and plasma markers of ADNC. In DLB, higher levels of p‐tau181 and p‐tau231 were associated with a lower risk of NPSs and hallucinations, indicating that ADNC is not a primary driver of NPSs in the early stages of DLB. Both elevated plasma p‐tau231 levels and the presence of hallucinations at baseline were independently associated with greater longitudinal cognitive impairment in DLB. Furthermore, we observed a significant interaction between these factors, suggesting that when hallucinations and AD pathology do co‐exist in DLB, it is particularly detrimental to cognitive function.

### 4.1 Association between p‐tau181 and p‐tau231 and NPSs in DLB and AD

In DLB, NPSs and hallucinations were more common in patients with lower markers of ADNC in vivo. Moreover, other in vivo studies have suggested that patients with “pure” DLB, without AD co‐pathology, have a higher prevalence of core features including visual hallucinations.^25^, ^53^ Similarly, clinicopathological studies have highlighted the role of LB pathology in the development of hallucinations in DLB throughout the disease course.^21^, ^30^, ^68^ The clinical heterogeneity associated with mixed LB‐AD pathology is well recognized, with accelerated cognitive decline and a worse overall prognosis.^24^, ^69^, ^70^ The LB‐AD phenotype likely contributes to the increased rate of misdiagnosis, and here we highlight the relatively lower preponderance of NPSs and hallucinations in DLB patients with AD co‐pathology in the early stages of disease.^69^ Characterizing the phenotype of patients with dual AD and LB pathology is important to improve diagnosis, and with the advent of new potentially disease‐modifying therapies, guide future clinical management.^54^, ^71^ The presence of NPSs early in the course of DLB may be an additional differentiator to support the enrichment of clinical trial populations.^52^ However, given that this is an observational study, we cannot ascribe mechanistic inferences to the inverse relationship between p‐tau and longitudinal NPSs in DLB. However, robust associations between LB and hallucinations have been reported previously,^29^, ^72^ suggesting it is LB rather than ADNC that is implicated in the etiology of hallucinations in early‐stage disease. To confirm this interpretation, future studies could explore the relationship between LB co‐pathology and NPSs in AD, both in post‐mortem studies and with the use of novel αSyn seed amplification assays in AD to identify the presence of co‐morbid synucleinopathy.

Increased concentrations of p‐tau231 and p‐tau181 at baseline were associated with a lower odds of developing hallucinations or NPSs in the following 2 years, highlighting their possible utility as predictive biomarkers characterizing the clinical phenotype. Higher plasma concentrations of p‐tau231, but not p‐tau181, were also associated with a lower risk of incident hallucinations and NPSs in patients without NPSs at baseline. P‐tau231 is a more sensitive marker of ADNC than p‐tau181 and reaches abnormal levels with a lower burden of more localized pathology.^47^ This may be of particular use to identify AD co‐pathology in DLB, where as a secondary pathology, distribution may be less widespread. Further studies are needed in DLB to explore the relative merit of the numerous p‐tau markers that are being developed.

Our findings contrast with clinicopathological studies that suggest that AD and LB pathologies contribute additively to a range of NPSs, including hallucinations.^21^, ^30^ However, post‐mortem studies primarily capture end‐stage disease, whereas in the current in vivo study the mean duration from the first symptom was 2 years.^29^, ^30^, ^32^ Furthermore, in sensitivity analyses, higher concentrations of p‐tau231 and p‐tau181 were associated with a lower risk of persistent hallucinations and NPSs in the early but not later stages of the disease. Thus AD co‐pathology may be associated with hallucinations later in the course of the disease where pathology is more widespread, akin to the late‐onset hallucinations reported in clinical studies of AD.^73^, ^74^ However, neuropathological studies with serial longitudinal clinical assessments prior to death are needed to characterize the evolution of NPSs associated with respective neuropathological changes.

In addition, our study highlights differences between neuropathological changes associated with hallucinations and delusions in AD and DLB. Hallucinations and delusions are often evaluated collectively as psychosis but increasingly evidence points to distinct neurobiological mechanisms with implications for their assessment and treatment.^75^, ^76^ Furthermore, the neurobiology of NPSs may differ across neurodegenerative diseases and within individual patients, possibly with different neuropathologies targeting similar neural networks leading to NPSs. This may contribute to some of the inconsistencies in associations reported between NPSs and AD pathology,^15^, ^77^, ^78^, ^79^ and this presents a challenge for future pharmacological treatments of NPSs, illustrating the need for a precision medicine approach.

The cross‐sectional nature of many studies is another factor that may contribute to variations across studies.^15^ When NPSs were measured longitudinally, we found that low concentrations of p‐tau181 or p‐tau231 at baseline were often associated with persistent but not single‐episode NPSs. Numerous additional factors such as psychosocial determinants also contribute to NPSs in dementia but are more likely to cause transient, single‐episode NPSs, which may be independent of underlying neurodegenerative disease. Longitudinal studies identifying persistent NPSs are likely to be more robust in identifying their neurobiological correlates and are likely to give greater insights as to the underlying pathology than cross‐sectional studies of NPSs.

### 4.2 Baseline NPSs and cognitive decline in DLB

In AD and PD, associations between NPSs and increased cognitive decline have been well established.^13^, ^74^, ^79^ However, fewer studies have explored this in DLB and one recent study found no association between NPSs and cognitive impairment in the prodromal stages of DLB.^59^ We found that hallucinations at baseline were associated with more significant cognitive impairment longitudinally, and this association was modulated by increasing levels of plasma p‐tau231. Furthermore, in a subgroup adjusted for education (*n* = 93), the presence of hallucinations at baseline was also associated with accelerated cognitive decline. Collectively this suggests that the presence of hallucinations in DLB has prognostic implications for cognitive function, particularly if p‐tau231 levels are elevated. Lewy body pathology in the limbic areas has been associated with both visual hallucinations and cognitive dysfunction in Parkinson's disease, suggesting there may be commonalities in the underlying mechanisms.^72^, ^80^, ^81^ Separately, plasma p‐tau231 is most strongly associated with tau deposition in the temporal and cingulate cortices and can differentiate individuals in the earliest Braak stages where neurofibrillary tangles deposit in the entorhinal regions, whereas plasma p‐tau181 is less sensitive to this early spread of tau.^46^, ^47^, ^49^ Therefore, it may be that the interaction between visual hallucinations and p‐tau231 level with cognitive dysfunction is a proxy for LB and AD pathology co‐localizing in the limbic and temporal regions and interacting to affect cognitive function, as is reported in neocortical regions.^82^ Where hallucinations and AD co‐pathology do co‐exist in DLB, it may indicate the presence of more extensive network involvement and neuropathological change driving cognitive dysfunction.^83^ It may also explain why no association between NPSs and cognitive impairment was found in the prodromal stages of DLB, where neuropathological change is likely to be more circumscribed, with fewer co‐pathologies and less pronounced cognitive dysfunction to validate associations.^59^, ^84^

### 4.3 Limitations

To our knowledge, this is the first study in DLB to evaluate associations between plasma markers of ADNC and NPSs, both cross‐sectionally and longitudinally. However, only one‐third of patients had biomarker confirmation of the diagnosis, and although the clinical diagnosis of probable DLB has high specificity, misdiagnosis is also reported.^62^, ^85^, ^86^ The retrospective, multicenter design of this study facilitated the inclusion of such a large DLB cohort but also involved some differences in the collection of data methods and instruments. One center did not use the NPI to assess NPSs and did not assess symptoms related to apathy or agitation; therefore data collected on these symptoms was for a smaller subgroup of patients. This smaller sample size may explain why we did not replicate associations found in clinicopathological studies between apathy, agitation, and ADNC. No information as to the ethnicity of participants included in the E‐DLB Consortium was available; this is a particular limitation in the context of DLB research, in which to date, representation across diverse populations has been very low.^87^ Furthermore, only three of five centers collected information regarding the education level of patients, a useful proxy of social determinants of health and cognitive reserve.

Although plasma p‐tau181 and p‐tau231 appear to be useful markers of ADNC in DLB, with good concordance with CSF and PET measures, universal cutoffs have not yet been established for the p‐tau assays used in this study due to substantial inter‐ and intra‐laboratory variation.^52^ Given the rapid progress of the field, consensus criteria point toward p‐tau217 being the reference blood marker for AD, which does have reported cutoffs and exists on fully‐automated platforms.^88^, ^89^ We did not include CSF or PET measures of ADNC in this study, which precludes definitive conclusions regarding the extent or distribution of ADNC in association with NPSs. Finally, in this study, we did not include other markers of pathology such as imaging markers of vascular change, the newer real‐time quaking‐induced conversion (RT‐QuIC) seed amplification assays of α‐synuclein, or specific but sensitive markers of neuronal damage such as neurofilament light (NfL), which have been associated with worse cognitive status and longitudinal trajectories in DLB.^90^, ^91^, ^92^ Future studies could identify additional co‐pathology with imaging and RT‐QuIC α‐synuclein assays, in addition to other markers of neurodegeneration and ADNC.^17^, ^52^

## 5 CONCLUSIONS

In this study, we found that patients with DLB, with a lower index of AD co‐pathology, had a greater longitudinal risk of NPSs and hallucinations. Early in the course of DLB, AD co‐pathology is not implicated in the etiology of NPSs and hallucinations, and NPSs appear less common in the early stages of disease in patients with mixed LB‐AD pathology. Hallucinations are associated with greater cognitive impairment in DLB, particularly where levels of plasma p‐tau231 are high. Further studies could explore the nature and localization of the interaction between AD co‐pathology and hallucinations with cognitive function.

## CONFLICT OF INTEREST STATEMENT

L.L.G., M.C.G., N.J.A., D.T.R., C.B., and A.L. have no conflicts to declare. A.P. has served on scientific advisory boards and/or as a consultant for Abbvie, Bial, Lundbeck, Roche, and Zambon Pharmaceuticals. C.P. has served on scientific advisory boards and/or as a consultant for EISAI, Lilly, Roche, Alzohiz, and Fujiribio. F.B. was the national coordinator for France for the Eisai Delphia (E2027) and Axovant Headway‐DLB therapeutic trials; he is currently the national coordinator for France of the Roche Graduate therapeutic trial; he had received honoraria from Roche, Eisai, and Biogen for oral presentations. H.Z. has served on scientific advisory boards and/or as a consultant for Abbvie, Acumen, Alector, Alzinova, ALZPath, Amylyx, Annexon, Apellis, Artery Therapeutics, AZTherapies, Cognito Therapeutics, CogRx, Denali, Eisai, LabCorp, Merry Life, Nervgen, Novo Nordisk, Optoceutics, Passage Bio, Pinteon Therapeutics, Prothena, Red Abbey Labs, reMYND, Roche, Samumed, Siemens Healthineers, Triplet Therapeutics, and Wave; has given lectures in symposia sponsored by Alzecure, Biogen, Cellectricon, Fujirebio, Lilly, Novo Nordisk, and Roche; and is a co‐founder of Brain Biomarker Solutions in Gothenburg AB (BBS), which is a part of the GU Ventures Incubator Program (outside the submitted work). D.A. has received research support and/or honoraria from Astra‐Zeneca, H. Lundbeck, Novartis Pharmaceuticals, Evonik, and GE Health, and has served as a paid consultant for H. Lundbeck, Axovant, Eisai, Heptares, Mentis Cura, Eli Lilly, and Biogen. Author disclosures are available in the Supporting Information.

## CONSENT STATEMENT

All participants gave written informed consent for the use of their de‐identified clinical and biomarker data.

## Supporting information



Supporting Information

Supporting Information
