# Supplementary material for: Plasma phosphorylated tau and neuropsychiatric symptoms in dementia with Lewy bodies
Source: Alzheimers Dement. 2024 Dec 28;21(2):e14434. doi: 10.1002/alz.14434 (PMC11848415; doi:10.1002/alz.14434)
Supplement: Supplementary file 2 — Supporting Information [file ALZ-21-e14434-s002.docx]

Supplementary Table 1. Baseline characteristics of subcohort with longitudinal assessment of NPS.

|  | DLB (n=172) | AD  (n=83) | p |
| --- | --- | --- | --- |
| *Demographic characteristics* |  |  |  |
| Age, y | 70.9 (8.9) | 72.0 (8.4) | 0.494 |
| Female, no. (%) | 79 (45.9) | 45 (54.2) | 0.231 |
| Education, y^a^ | 9.5 (4.3) | 10.2 (2.3) | 0.474 |
| Duration of symptoms, y^b^ | 1.88 (2.7) | 0.67 (2.0) | **<0.001** |
| MMSE score | 23.9 (4.8) | 23.7 (4.1) | 0.342 |
| *Neuropsychiatric symptoms* |  |  |  |
| Hallucinations | 94 (54.7) | 11 (13.8) | **<0.001** |
| Visual hallucinations or illusions^c^ | 53 (51.5) | 9 (13.2) | **<0.001** |
| Auditory hallucinations^c^ | 16 (15.5) | 0 | **<0.001** |
| Olfactory hallucinations^c^ | 16 (15.7) | 2 (2.9) | **0.010** |
| Delusions | 33 (21.2) | 7 (9.2) | **0.026** |
| Depression | 57 (38.8) | 16 (21.9) | **0.015** |
| Agitation^d^ | 14 (20.6) | 5 (41.7) | 0.144 |
| Apathy^d^ | 29 (43.3) | 7 (58.3) | 0.364 |
| Any NPS | 135 (78.5) | 26 (32.1) | **<0.001** |
| *Plasma biomarker* |  |  |  |
| Plasma ptau181 level, pg/ml | 17.7 (10.0) | 21.2 (9.3) | **<0.001** |
| Plasma ptau231 level, pg/ml | 12.4 (6.0) | 14.7 (6.5) | **0.002** |

DLB, dementia with Lewy bodies; AD, Alzheimer’s disease; HC, healthy control; MMSE, Mini Mental State Exam; NPS, neuropsychiatric symptom. ^a^n=62 DLB, n=10 AD) ^b^n=147 DLB, n=63 AD), ^c^assessed in one centre: n=103 DLB, n=68 AD); ^d^assessed in 4 centres n=68 DLB, n=12 AD)

**Supplementary table 2. Adjusted logistic regression model with odds ratio of cross-sectional NPS with ptau181 and ptau231 separately for DLB and AD.**

| *Logistic Model for DLB* |  |  |  |  |  |  |  |  |  |
| --- | --- | --- | --- | --- | --- | --- | --- | --- | --- |
|  | **Ptau181** | | | **Ptau231** | | | Total Sample | |  |
| **Symptom** | **OR** | **95% CI** | **p-value** | **OR** | **95% CI** | **p-value** |  |  |  |
| Hallucination | 0.76 | 0.55 - 1.07 | 0.115 | 0.82 | 0.59 - 1.16 | 0.259 | 175 | 176 |  |
| VH or illusions | 0.38 | 0.21 - 0.70 | 0.020 | 0.59 | 0.36 - 0.97 | 0.038 | 85 | 85 |  |
| Delusions | 1.16 | 0.85 - 1.58 | 0.359 | 1.42 | 0.99 - 2.04 | 0.057 | 162 | 163 |  |
| Depression | 1.35 | 0.98 - 1.85 | 0.066 | 1.22 | 0.90 - 1.65 | 0.202 | 155 | 156 |  |
| Agitation | 1.24 | 0.75 - 2.05 | 0.394 | 1.55 | 0.90 - 2.68 | 0.116 | 89 | 90 |  |
| Apathy | 1.19 | 0.80 - 1.78 | 0.397 | 0.86 | 0.53 - 1.38 | 0.530 | 89 | 89 |  |
| Any NPS | 0.73 | 0.52 - 1.01 | 0.058 | 0.77 | 0.55 - 1.08 | 0.126 | 175 | 176 |  |
|  |  |  |  |  |  |  |  |  |  |
| *Logistic Model for AD* |  |  |  |  |  |  |  |  |  |
|  | **Ptau181** | | | **Ptau231** | | |  |  |  |
| **Symptom** | **OR** | **95% CI** | **p-value** | **OR** | **95% CI** | **p-value** |  |  |  |
| Hallucinations | 1.05 | 0.50 - 2.18 | 0.904 | 0.78 | 0.42 - 1.44 | 0.429 | 93 | 93 |  |
| VH or illusions | 1.00 | 0.34 – 2.96 | 0.999 | 0.48 | 0.32 - 1.75 | 0.498 | 47 | 47 |  |
| Delusions | 2.05 | 1.11 - 3.78 | 0.022 | 1.31 | 0.55 - 3.09 | 0.541 | 90 | 90 |  |
| Depression | 2.06 | 1.17 - 3.63 | 0.012 | 1.56 | 0.89 - 2.75 | 0.119 | 87 | 87 |  |
| Agitation | 1.17 | 0.53 - 2.58 | 0.691 | 1.19 | 0.52 - 2.68 | 0.683 | 46 | 46 |  |
| Apathy | 1.34 | 0.61 - 2.97 | 0.465 | 1.48 | 0.71 - 3.12 | 0.299 | 46 | 46 |  |
| Any NPS | 1.38 | 0.86 - 2.22 | 0.181 | 1.24 | 0.79 - 1.95 | 0.343 | 94 | 94 |  |
